# Reflections on Addiction in Students Using Stimulants for Neuroenhancement: A Preliminary Interview Study

**DOI:** 10.1155/2015/621075

**Published:** 2015-05-03

**Authors:** Elisabeth Hildt, Klaus Lieb, Christiana Bagusat, Andreas G. Franke

**Affiliations:** ^1^Center for the Study of Ethics in the Professions, Illinois Institute of Technology, 3241 S. Federal Street, Chicago, IL 60616, USA; ^2^Department of Philosophy, University of Mainz, Jakob Welder-Weg 18, 55099 Mainz, Germany; ^3^Department of Psychiatry and Psychotherapy, University Medical Centre, Untere Zahlbacher Straße 8, 55131 Mainz, Germany; ^4^Department of Social Work and Education, University of Neubrandenburg, University of Applied Sciences, Brodaer Straße 2, 17033 Neubrandenburg, Germany

## Abstract

The use of stimulants for the purpose of pharmacological neuroenhancement (NE) among students is a subject of increasing public awareness. The risk of addiction development by stimulant use for NE is still unanswered. Therefore, face-to-face interviews were carried out among 18 university students experienced in the nonmedical use of methylphenidate and amphetamines for NE assessing aspects of addiction. Interviews were tape-recorded, verbatim-transcribed, and analyzed using a qualitative approach. The interviews showed that participants—the majority had current or lifetime diagnoses of misuse or addiction to alcohol or cannabis—reported an awareness of the risk of addiction development associated with stimulant use and reported various effects which may increase their likelihood of future stimulant use, for example, euphoric effects, increase of self-confidence, and motivation. They also cited measures to counteract the development of addiction as well as measures taken to normalize again after stimulant use. Students were convinced of having control over their stimulant use and of not becoming addicted to stimulants used for NE. We can conclude that behavior and beliefs of the students in our sample appear to be risky in terms of addiction development. However, long-term empirical research is needed to estimate the true risk of addiction.

## 1. Introduction

The use of “smart drugs” containing over-the-counter- (OTC-) drugs as well as prescription drugs and illicit drugs for the purpose of pharmacological neuroenhancement (NE) by healthy people has attracted an increasing amount of attention [1–4]. In particular, (psycho-) stimulants such as methylphenidate (MPH) and amphetamines (AMPH) such as prescription amphetamines (e.g., Adderall, attention) as well as illicit amphetamines (e.g., ecstasy, speed) seem to be the most prevalent substances used for the purpose of enhancing mental performance as previous quantitative surveys prove: Among university students, prevalence rates range broadly from 1 to 38% [5–9].

In interdisciplinary debates on medical, social, and ethical implications of NE, aspects relating to addiction play a considerable role [10–14]. The common mode of action of AMPH and MPH is an interaction with synaptic norepinephrine and dopamine transporters. In contrast to MPH, AMPH additionally leads to vesicular release (exocytosis) of dopamine, which means an increase of action-independent dopaminergic activity [15–17].

Based on theoretical reflections on the mechanisms of action of NE drugs, Heinz and colleagues (2012) argue that any current or future NE drug will have the potential to induce dependence; given this risk of addiction, for ethical reasons they argue against clinical research into NE [10]. This position is rejected by Shaw [12] who argues that safety and research in the area of NE do not pose unique difficulties.

Smith and Farah point out that, according to an estimate by Kroutil and colleagues (2006), 5% of the nonmedical users of prescription stimulants meet the criteria for abuse or dependence. However, the immediate and long-term risk of abuse or addiction among stimulant users for NE is unclear [14, 18].

Although no data is available on the percentage of addicted subjects among stimulant users for NE, the addiction risk of stimulants is well known among students: An interview study by Partridge and colleagues among healthy university students without experiences of stimulant use for NE revealed that they identified psychological dependence as a potential negative consequence [19]. In our own interview study of 18 student users of MPH and AMPH, we also demonstrated students' concerns about the risk of addiction regarding stimulants compared to caffeine [20] and found that users of stimulants for NE showed significantly higher rates of misuse of alcohol and cannabis compared to healthy controls [21].

In view of the medical data indicating that stimulants used for NE come with the risk of dependence and given the fact that many who have heard about NE believe that it might induce dependence, the aim of this study was to describe students' behaviour with respect to stimulant use and dependence with the help of semistructured interviews with students who have taken stimulants for NE.

## 2. Methods

By posting placards on public bulletin boards throughout the campus of the University of Mainz from 2009 to 2010 we searched for healthy participants (without any psychiatric disorders leading to the necessity of being prescribed stimulants such as MPH or AMPH) who had already used prescription or illicit stimulants (AMPH, MPH, ecstasy, and cocaine) for the explicit purpose of NE. Of the thirty students who contacted us via telephone or e-mail, only twenty-two responded to our e-mail requests for an appointment and agreed to participate. Twenty-two interviews were carried out. Two students had to be excluded because of having current physicians' prescriptions for stimulants (e.g., Ritalin); two interviews failed for technical reasons (the recorder did not work appropriately). Finally, eighteen interviews entered further analysis.

In previous studies we already analyzed the interview transcripts of these 18 users regarding moral differences between illicit stimulants and caffeine (legal stimulant drug) and regarding life context of pharmacological academic performance enhancement [20, 22]. In addition, data about a general overview of knowledge about stimulants for CE, patterns of use, and diagnoses of abuse and addiction compared to twenty healthy controls have already been published [21].

Each interview was divided into two parts. First, a trained psychologist conducted a structured clinical interview (SKID). This interview is a diagnostic instrument to indicate major mental disorders and personality disorders. The SKID and questions on psychoactive medication as a result of having a psychiatric disorder with the need of psychoactive medication ensured that potential participants with psychiatric disorders and having psychoactive medication with a physician's prescription for medical reasons could be excluded from the analysis. Addiction was no reason for study exclusion. Second, based on a semistructured interview guideline, two interviewers asked open and closed questions regarding the nonmedical use of substances for CE. Beyond sociodemographic data such as age, area of study, and grades, we asked: “Do you think that the/your use of illicit stimulants may lead to addiction?”, “Which effects of the stimulant you used did you experience?”, and “Have you experienced a need to increase dosage in order to achieve the same level of effects?” In addition, interviewees had the opportunity to tell us about further aspects important to them.

Prior to the interview, participants gave written informed consent for being interviewed and for tape-recording. Each participant received thirty Euros for compensation after he/she had been interviewed. The local Ethics Committee (Landesärztekammer Rheinland-Pfalz, Medical Association, Rheinland-Pfalz) approved this interview study.

**Figure 1:**
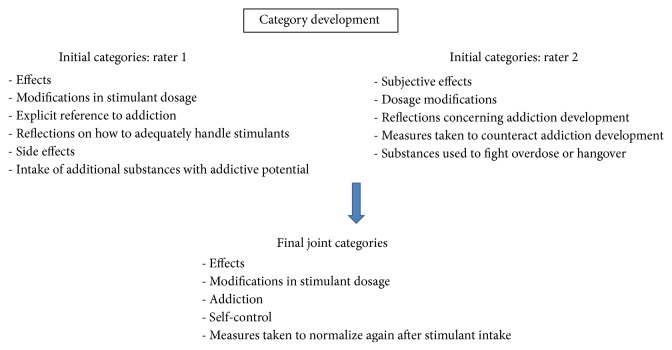
Category development.

One independent person transcribed the contents of the tape-recorded interviews. Afterwards, transcriptions were analysed by two raters with a qualitative approach based on inductive category development [23, 24]. The two raters analyzed the transcripts independently. Rater 1 came up with 6 initial categories and rater 2 with 5 initial categories (cf.  Figure 1). The raters discussed the initial categories and developed a joint set of 5 categories. Some of the categories directly resulted from the respective interview questions. Because of a high level of agreement during the category discussions, no third rater was needed.

## 3. Results

Among all eighteen participants (100% who met the inclusion criteria and whose interviews were correctly tape-recorded and analysed), fourteen had used illicit AMPH and eight prescription MPH. Four student participants (22.2%) used both prescription and illicit stimulants for academic NE.

For participants' characteristics see Table 1 and one of our previous publications [20]. As described in our previous study on these subjects, SKID interviews revealed that the vast majority (88.9%, 16 of 18 participants) of the participants had current or lifetime diagnoses of misuse or addiction of alcohol, cannabis, or AMPH (see Table 2) [21].

We analysed contents of the interviews associated with addiction-related issues. In order to present the answers we obtained in more detail, we grouped the answers as follows: (1) effects, (2) modifications in stimulant dosage, (3) participants' evaluation of aspects of addiction, (4) self-control, and (5) measures taken to counteract stimulants' effects and to “normalize” again after stimulant use.

### 3.1. Effects

All student participants had used stimulants with the intention of NE. Each participant described the subjective effects of stimulant use in a slightly different way. The answers included rather general descriptions of positive cognitive, motivational, and emotional effects such as having increased alertness, being fitter, being in a better mood, experiencing an increase in self-confidence, being more communicative, being better focused, having better concentrated attention, being better motivated, being more vigilant, being more cheerful, being more energetic, experiencing euphoria or euphoric episodes, feeling strengthened, or feeling ready to take on anything. Others said that stimulant use would lead to being less able to respond to criticism, being less sensitive but more automatic, feeling closer to oneself, being a little bit hysterical or hyped up, being more aggressive, being more distressed, experiencing an increased tendency to do one's own thing, feeling detached, or experiencing an increase in appetite.

In sum, the effects recognized after the use of stimulants for NE described by the participants are interwoven with aspects which favor—at least in parts—the development of addiction.

### 3.2. Modifications in Stimulant Dosage

In the interviews, we asked the participants whether during the time period when they used stimulants for NE there had been a need to increase the dosage of stimulants in order to maintain the effects. Three participants said yes, whereas twelve answered that there was no need to increase dosage; three were ambivalent regarding dosage.

One of those students who increased dosage said: “At least I felt as if the effect reduced. […] But I don't know if that actually happened.”

According to the answers obtained, the need to increase dosage depends on the method and duration of stimulant intake. Whereas one student stressed that in view of the short duration of intake there was no need for dosage increase, another participant said with regard to AMPH use: “If you take it several days in a row, then you have to increase the dose in order to reach the same effect. Otherwise if there are more than two weeks in between, it's always the same dose.” Another student stated that he knew from others about the perceived need to increase dosage and that in view of this he carefully paid attention not to increase dosage: “I really keep an eye on not having to increase the dose, also because I don't want to do so.”

### 3.3. Addiction

When asked whether they believe that the use of stimulants for NE may lead to addiction eleven participants responded “quite likely.”

For several participants, the risk of addiction played a considerable role. One student said that the experience of stimulants providing support in one's daily routine might facilitate addiction: “A certain addiction can definitely come up very quickly because it helps someone in their everyday life and with all these pressures to perform an addiction can be expected.”

Some of the interviewees distinguished between physical and mental addiction. For most participants, physical aspects of addiction do not seem to be of primary relevance. In contrast, several participants stressed mental aspects of addiction.

One student talked about his impression that high expectations concerning stimulant effects that are not totally fulfilled by subsequent uses may facilitate dependency.

“When I take something for the first time it works perfectly, the second time the effect is actually the same but you already expect it to work as well. But if you already approach it like that, I feel as if the effect was very good the second time, but subjectively a tiny little bit worse than the first time.”

### 3.4. Self-Control

In spite of the fact that a great number of the participants believed that NE substances have some addictive potential, most of them felt that they have things under control. In order to underline this, some interviewees reported having taken measures to counteract the risk of addiction. Several students said that for them it is important to have nonconsumption intervals in between in order to prevent the development of addiction. For example: “Because I wanted to maintain the feeling that I only take the substances when I want to and not because I have to [due to an addiction], I've been taking an abstinence break for almost three months.”

Another one said that he introduced nonconsumption intervals “in order to avoid enjoying it too much.”

One interviewee stressed reasonableness and pointed out that he is using stimulants very deliberately:

“I do all that very carefully and not because of any addiction. I never got addicted to anything. […] I always watch out where I get the stuff from.”

Another student explicitly mentioned individual responsibility in this context. He said:

“I think it's an important aspect that you can learn how to handle it. And that you can do it in a responsible way. That's why you should only do it occasionally. To me that's one main aspect.”

### 3.5. Measures Taken to Normalize Again after Stimulant Intake

Several participants reported negative effects and problems that occurred following the duration of stimulating effects after stimulant intake. These after-effects include sleeplessness and heightened alertness, but also feelings of depression, lack of energy, and sleep problems.

Some of the interviewees said that, in order to avoid problems related to sleeplessness and heightened alertness that may arise afterwards, they took special measures.

One of them said: “I was a serious chain smoker and drank loads of alcohol in order to calm myself down a little bit.”

Other interviewees also talked about illegal drugs and prescription drugs:

“After the last consumption you have to stay awake quite a while until you're tired enough to fall asleep. There is the opposite consumption, you can drink loads of alcohol that works against it, or some smoke weed in order to calm down to then fall asleep.”

Another example is: “When I took a really high dose during the day, I took [Zoplicone, Tetrazepam, or Melatonin] to fall asleep. That's pretty strong, which is actually the reason why I don't take it every day.”

Another interviewee reported “I always have diazepam at home, which is an antidote for most drugs.”

## 4. Discussion

In the present study, we focused on aspects of addiction among students who used stimulants for NE purposes. Students talked about several aspects of misuse and addiction including desirable effects, dosage of the stimulants used, self-control, and means taken to counteract stimulant effects and to “normalize” again after stimulant use. It is important to stress that the data obtained in this preliminary qualitative study is in no way representative and that, based on the spontaneous answers of the interviewees, we are not able to draw any definitive conclusion on the effects of stimulants for NE in healthy individuals.

Furthermore, the student participants are a self-selected group. In order to avoid attracting students with an urgent need of money, we choose to offer a low to moderate remuneration of thirty Euros. Thus, we do not think that the recruitment process favoured participants with current drug addictions.

The average age of the interviewees of 25.8 years is very high, and two-thirds are male. Possible reasons for this may be that older students experience an increase in pressure to graduate soon or that those who take longer time to finish their studies may tend to seek additional distractions and experiences. It may also be speculated that male students are more prone to experiment. The results obtained are in accordance with data collected by DeSantis and Hane [25], who reported a higher percentage of male students using stimulants for enhancement and a higher incidence in older students. The number of students interviewed is too low to draw any conclusion on whether in certain fields of study a higher percentage of students misuse stimulants than in others. During the period of study recruitment (2009-2010), there have not been any major changes in the university setting that impacted the students' use of stimulants.

In the International Classification of Diseases version 10 (ICD-10) stimulant dependence is described in the ICD-10 F 15.2. Symptoms to diagnose stimulant dependence include the presence of physical and psychological damage, craving, reduced ability to control stimulant use, development of stimulant tolerance with increased dosage for stable stimulant effects, withdrawal symptoms in case of abstinence, and mentally focusing on stimulant use and continued use despite detrimental consequences of stimulant use. In the Diagnostic and Statistical Manual of Mental Disorders (version DSM-IV-TR), AMPH dependence can be found in chapter 304.40 and is diagnosed when an individual persists in AMPH use despite problems related to use of the AMPH; compulsive and repetitive use may result in tolerance to the effect of AMPH and withdrawal symptoms when AMPH use is reduced or stopped. The results of this study have to be considered in view of these diagnostic criteria.

All stimulant-using participants of this study aimed at NE. Beyond that, most of the described types of effects show “pleasant” states that increase the likelihood of using the stimulant again. Among the most significant ones are an increase in self-confidence, euphoric episodes, being more communicative, being fitter, being in a better mood, being better motivated, more cheerful, more energetic, feeling strengthened, and feeling ready to take on anything.

A very important aspect for the diagnosis of addiction according to the ICD-10 and DSM-IV-TR is an increase of dosage because of tolerance development and the perceived need to increase dosage in order to maintain the desired level of stimulating effects. At least in some consumers, stimulant use went along with some adaptation process. Three out of 18 participants said that, during the overall time span of stimulant consumption, there was a need to increase the dosage, and three were ambivalent. It is important to stress that, for those who used stimulants during a longer period of time, the answer to this question might be indicative of some addictive tendency. In contrast, the question concerning dosage increase does not apply to those who used stimulants only once or a few times. The users were well aware of this context. In several interviews, the wish to avoid an increase in dosage was picked out as a central theme.

The majority of the users believed that stimulant use for NE quite likely or definitely may lead to addiction. This is in accordance with the results obtained in our quantitative survey among high school students and university students [26], according to which more than 90% of the participants believed that NE drugs could lead to addiction.

With regard to the perceived risk of addiction, for the interviewees frequency of use clearly played a role which is in line with the reflections by Compton and Volkow [13]: The perceived risk of addiction was one of the reasons why several users reported on having introduced longer intervals in stimulant administration. Others said that they assume that the risk of addiction would be higher the longer a person uses stimulants for NE.

Concerning the risk of addiction, the students stressed mental aspects of addiction, whereas they did not consider the risk of physical addiction to be considerable. Strikingly, with regard to a perceived risk of addiction, several students confidently talked about having control over the situation. They reported intentionally having introduced intervals in which they did not take stimulants in order to avoid becoming addicted, or they cited other measures taken to avoid having to increase the dosage. Several users asserted that they control the situation and that the substance does not control them, be it by some addictive potential or by the perceived need to use it. The underlying ideas seem to be as follows: I am strong enough to withstand becoming addicted and I want to autonomously decide on whether or not to take the drug.

Apart from the participants' subjective beliefs regarding their self-control of the use of MPH and AMPH for NE, objective diagnoses using SKID interviews show very high rates of current or lifetime misuse and addiction with regard to alcohol, cannabis, and AMPH on the one hand. On the other hand, there were only one participant diagnosed with current AMPH misuse and no participant diagnosed with current AMPH dependence. It is important to stress, however, that we do not know whether the reason for current lack of AMPH dependence and scarce diagnoses of current AMPH misuse is due to the fact of efficient self-control or the fact that the participants currently do not use any stimulants for NE. Therefore, more research is needed to analyze the risk of developing stimulant misuse and/or dependence in the context of using stimulants for NE.

In the interdisciplinary debate on NE, ethical issues concerning risks and benefits, fairness, justice, cognitive liberty, autonomy, authenticity, and personal identity are intensively discussed [27]. Individual autonomy is one of the key concerns; such concerns imply that it is up to the individual person to decide freely on whether or not to modify one's brain chemistry [28, 29]. NE drugs' addictive potential may curtail individual autonomy in a highly problematic way, for addiction clearly undermines a person's capacity to autonomously decide on whether or not to use NE drugs [30, 31]. The users in our study clearly experience this threat to autonomy.

Heinz et al. (2012) suggest that any risk of addiction associated with the use of stimulants for NE will result in a very unfavourable risk-benefit ratio for these kinds of NE and will be a central ethical argument against running clinical trials on NE [10]. Despite their acknowledgement of the risk of addiction, a considerable part of the interviewees nevertheless continued using these substances. One reason for their continued use may be the self-reassuring strategies described above and their impression of having control over the situation. The putative subjective benefits of NE, which may lead participants to accept the risk-benefit ratio, serve as another explanation.

In any case, in spite of the risks involved, the users voluntarily enroll in some kind of self-experiment on the effects and side effects of NE. Some users stressed individual responsibility and argued that they use stimulants for NE in a responsible way as long as they have control over the situation and avoid getting addicted or having unforeseen side effects. These attitudes lead us to the question: Is pharmacological NE something that can be done in a responsible way? It seems that at least some NE consumers do assume this.

In sum, the risk of addiction and the need to find an adequate strategy in order to prevent addiction matter considerably to the interviewees. This is in accordance with Hall and Lucke who noted that MPH is under “legal control because of the high rates of dependence and adverse effects experienced by regular users” [32]. According to Forlini and Racine, as well as Partridge and colleagues, beneficial effects of stimulants for NE are widely portrayed in the media while the risk of dependence is underreported [33, 34]. The results obtained in this study give a clear hint to the need to pay more attention to the addictive potential of stimulants used for NE.

In addition, the after-effects of stimulant intake have to be taken into consideration. Here, the users reported on a broad spectrum of effects from heightened alertness and sleeplessness to depressive periods and lack of energy. Some users reported on having taken measures after stimulant intake to “normalize,” which include drinking alcohol, smoking cannabis, or taking benzodiazepines. Such consumption carries the potential for further individual and health problems, such as polytoxicomania. Furthermore, these extra measures to normalize point to the negative influence that coingestion of other drugs may have the risk of developing dependence. Taken together, in order to adequately consider the effects of drug use for NE on the individual persons involved, it is important to take into account the pharmacological after-effects as well as additional drugs used in order to normalize again.

## 5. Conclusion

In the present interview study, the student participants used stimulants with the aim of NE. The risk of addiction by stimulant use played a considerable role in the answers obtained. Taken together, the subjects reported on a broad spectrum of aspects of addiction related to stimulants used for NE, including an awareness of the fact that stimulants imply a potential risk of addiction. Even if the participants are well aware of this fact, they evaluate the risk as being considerably low for themselves. Furthermore, several interviewees talked about strategies that serve as a means to control stimulant use and to counteract stimulant dependence. Behavior and beliefs of the students in our sample appear to be risky in terms of addiction development. However, long-term empirical research is needed to estimate the true addiction risk of stimulant use for NE and to characterize factors associated with a high risk of becoming addicted.

## Figures and Tables

**Table 1:** Characteristics of participants.

Characteristics	Percentage/number 100%, *n* = 18
Gender	66.7% male (*n* = 12)
	33.3% female (*n* = 6)
Age (mean ± SD)	25.8 years ± 2.88
Completed semesters (mean ± SD)	7.35 semester ± 3.79
Department of	
Humanities	44.4% (*n* = 8)
Natural sciences	33.3% (*n* = 6)
Economics	22.2% (*n* = 4)

Data are given as mean ± standard deviation (SD) according to Franke et al. [20].

**Table 2:** Diagnoses of misuse and dependence among all interviewed student participants with the use of a structured clinical interview (SCID-I).

Diagnoses of substance misuse and dependence (total number of users: *n* = 20)	Lifetime (past) diagnoses		Current diagnoses	
Alcohol misuse	*n* = 9	45%	*n* = 7	35%
Alcohol dependence	*n* = 3	15%	*n* = 2	10%
Cannabis misuse	*n* = 5	25%	*n* = 1	5%
Cannabis dependence	*n* = 4	20%	*n* = 1	5%
Amphetamine misuse	*n* = 2	10%	*n* = 1	5%
Amphetamine dependence	*n* = 2	10%	*n* = 0	0%

Originally 20 student participants had been surveyed by interview questionnaires [21]. 18 were tape-recorded and verbatim-transcribed for further analysis with a qualitative approach based on inductive category development [23]. Table 2 contains all diagnoses regarding substance misuse and dependence; there were no diagnoses of misuse or dependence of further substances.
